# Physical and Mental Components of Quality of Life after a Cardiac Rehabilitation Intervention: A Systematic Review and Meta-Analysis

**DOI:** 10.3390/jcm13185576

**Published:** 2024-09-20

**Authors:** José Moreira, Jorge Bravo, Pedro Aguiar, Bruno Delgado, Armando Raimundo, Paulo Boto

**Affiliations:** 1 Escola Superior de Enfermagem São João de Deus, University of Évora, 7000-811 Évora, Portugal; 2 Comprehensive Health Research Centre (CHRC), University of Évora, 7000-849 Évora, Portugal; jorgebravo@uevora.pt (J.B.); ammr@uevora.pt (A.R.); 3 Escola de Saúde e Desenvolvimento Humano, University of Évora, 7000-849 Évora, Portugal; 4 National School of Public Health, NOVA University of Lisbon, 1600-560 Lisbon, Portugal; pedroaguiar@ensp.unl.pt (P.A.); paulo.boto@ensp.unl.pt (P.B.); 5 Institute of Health Sciences, Catholic University of Portugal, 1649-023 Lisbon, Portugal; bruno.m.delgado@gmail.com

**Keywords:** patient-reported outcome measures, cardiac rehabilitation, health-related quality of life, randomized controlled trial, meta-analysis

## Abstract

**Background:** This study aimed to analyze the effect of cardiac rehabilitation programs on the health-related quality of life of patients after a coronary cardiac event using patient-reported outcome measures (PROMs) for up to 6 months of evaluation. **Methods**: A comprehensive search was carried out in the MEDLINE, CINAHL, CENTRAL, and Web of Science databases for randomized controlled trials comparing the cardiac rehabilitation program with usual care. Two independent reviewers assessed the studies for inclusion, risk of bias using the Cochrane tool, and quality of evidence through the GRADE system. A meta-analysis was performed on studies assessing health-related quality of life with the SF-12 (Physical Component Summary and Mental Component Summary) up to 6 months after the program. **Results**: Twelve studies encompassed 2260 patients who participated in a cardiac rehabilitation program after a coronary event, with a mean age of 60.06 years. The generic PROMs used to assess quality of life were the SF-12, SF-36, EQ-5D-3L, EQ-5D-5L, and GHQ, and the specific coronary heart disease PROMs were MacNew and HeartQoL. There was a positive effect of participation in cardiac rehabilitation on the physical component of health-related quality of life at 6 months (MD [7.02]; *p* = 0.04] and on the mental component (MD [1.06]; *p* = 0.82) after applying the SF-12. **Conclusions**: This study highlights the significant benefits of cardiac rehabilitation programs on health-related quality of life, particularly in the physical domain at 6 months. Assessing outcomes over time through PROMs after coronary heart events is essential, thus making it possible to personalize patients’ care and improve their health status.

## 1. Introduction

Cardiovascular disease is a major public health problem associated with high mortality and morbidity, reduced exercise capacity, and lower quality of life [1]. It is particularly important to consider risk factors since the risk for cardiovascular disease combines the direct cause of disease with concepts of probability, prediction, and prognosis [2]. A cardiac rehabilitation (CR) program consists of a multidisciplinary intervention after a coronary cardiac event. This multidisciplinary intervention is aimed at lifestyle changes, smoking cessation, blood pressure control, adequate weight, a balanced diet, and encouraging physical activity [3]. To change lifestyles and control and optimize the prognosis of cardiac disease, and according to the guidelines reported by scientific societies, CR programs are recommended to achieve and maintain optimal health in these patients [3]. CR programs have grown and evolved in preventing and treating cardiovascular disease, demonstrating effectiveness, and contributing to participants’ quality of life [4,5]. Therefore, quality of life can be improved through psychosocial support facilitating a return to work, increased health literacy optimizing healthy behaviors, and encouraging physical activity to improve cardiac function and reduce anxiety levels.

Patient-reported outcome measures (PROMs) are used to assess the health-related quality of life (HRQoL) of patients, showing their health status and functional capacity and allowing for informed clinical and health decisions [6,7,8]. The assessment instruments used to standardize PROMs can be generic or specific [9]. Generic instruments make it possible to assess a particular pathology in the general population, while specific instruments assess the results of a particular population, making it possible to test the effectiveness of treatments for the same disease [9]. Given the quality of outcomes that PROMs can offer and individualized care from the patient’s perspective, there is growing interest in using PROMs. In this emerging area of research, the interest in studying PROMs in patients with cardiac pathology and the impact they may have on optimizing CR programs is noticeable [7].

However, despite the demonstrated benefits, there is a lack of comprehensive reviews focusing on the longitudinal impact of CR programs on both the physical and mental components of HRQoL. Some studies have already been conducted in this area, showing a positive effect on the quality of life of patients participating in this type of program through the assessment made using PROMs [10,11]. Other studies have demonstrated that HRQoL improved after participating in CR programs [12] or physical exercise programs [13]. However, some of these studies only used a physical intervention and did not address the mental and physical components according to the duration of the program and the type of PROM. Given this lack of knowledge and the importance of PROMs in assessing the effect of a CR program on HRQoL at 6 months [11], this systematic review with meta-analysis was carried out with the following aim: analyze the effect of CR programs (phase II) on the HRQoL of patients after a coronary cardiac event using PROMs for up to 6 months of evaluation. This study is important for the scientific and clinical community, as it allows us to understand the effect of CR on the physical and mental dimensions of the quality of life perceived by patients up to 6 months after an acute coronary event.

## 2. Methods

The protocol of this systematic review with meta-analysis was registered in the International Prospective Register of Systematic Reviews (PROSPERO) with the number CRD42022344240. A systematic review of peer-reviewed English language articles was undertaken according to the recommendations of the Preferred Reporting Items for Systematic Reviews and Meta-Analysis (PRISMA) group [14], as shown in the PRISMA checklist.

### 2.1. Research Question and Eligibility Criteria

The research question addressed in this study was as follows: What is the effect of CR programs on mental and physical components after a coronary cardiac event for up to 6 months of assessment with PROMs?

We included all RCTs comparing interventions in CR programs against usual care, focusing on assessing HRQoL through PROMs. We included studies comparing the group of adult participants with coronary cardiac events who took part in a CR intervention (telephone or supervised follow-up, health education, structured exercise, and dietary support) with control groups that received guideline-recommended medical treatment. These CR programs are comprehensive, including a supervised or unsupervised structured exercise component, with educational support on risk factors and pharmacological therapy, and informal follow-up or education on exercise, psychosocial support, and diet. The comparison group received usual care (a non-standard, unstructured intervention without follow-up by specialist health professionals). In addition to these aspects that make up the programs, those in which HRQoL was assessed through PROMs up to 6 months after the start of the program were considered.

### 2.2. Search Strategy and Selection of Studies

Comprehensive searches were conducted in MEDLINE via Ovid, CINAHL via EBSCO, the Cochrane Central Register of Controlled Trials (CENTRAL), and Web of Science (SciElo) from January 2017 up to September 2024. This period was set for the research because in 2016 the European guidelines on the prevention of cardiovascular diseases in clinical practice were updated [2], which has an impact on studies with results based on CR programs. For the development of this search strategy, we used the Mesh Medical Subject Heading (MeSH) terms and MeSH major topic, following the methods suggested by the Cochrane Handbook for Systematic Reviews of Interventions [15]. Studies that met the criteria defined by the PICO(D) framework and that reported data on outcomes related to the implementation of the CR program were included. The studies were selected by two reviewers, following the eligibility criteria: (1) adult patients with coronary heart disease; (2) CR program phase II—patients after coronary cardiac event; (3) usual care after coronary cardiac event treatment with assessment of HRQoL over time (baseline and follow-up during or after intervention); (4) generic PROMs for HRQoL assessment and specific PROMs to coronary heart disease; (5) randomized clinical trials. The exclusion criteria were as follows: (1) abstracts; (2) studies not in English; (3) non-explicit assessment moments with PROMs; (4) non-original studies (reviews and meta-analyses, study protocols, conference proceedings, letters, commentary, and reports). Grey literature was also searched, and the references of the included studies were reviewed, with the aim of making important contributions to our study and increasing the scope of this systematic review.

### 2.3. Data Extraction

After considering the inclusion criteria, software was used to remove duplicate articles and manage the articles included for the final review. After removing the duplicates, two authors independently screened all the titles and abstracts for possible inclusion according to the predefined eligibility criteria. Disagreements were resolved by consensus or by consulting a third author (an expert in CR) when needed. After reviewing the titles and abstracts, a full-text analysis was conducted.

### 2.4. Risk of Bias

Version 2 of the Cochrane risk-of-bias tool for randomized trials (RoB 2) was used for this study. The evaluation was specific to the outcome of HRQoL for the effect of an intervention on the experimental and control groups. Through this tool structured in 5 domains, it was possible to evaluate the different types of bias that can affect the results of randomized trials. These are bias arising from the randomization process; bias due to deviations from intended interventions; bias due to missing outcome data; bias in measurement of the outcome; and bias in the selection of the reported result. The risk-of-bias assessment for each of the included studies was performed by one investigator then reviewed and validated by the second investigator (J.B.), and doubts were discussed in a final meeting.

### 2.5. Quality of Evidence Assessment

Evidence levels were evaluated using the Grading Recommendations Assessment, Development, and Evaluation (GRADE) methodology [16] for primary outcomes at 6 months of the CR program. The certainty of the evidence was classified as high, moderate, low, or very low, with randomized clinical trials considered as high-certainty evidence.

### 2.6. Data Synthesis and Analysis

The analysis of the different studies included the following information: author and year, method, sample size, exercise type, duration, intervention, and outcome measures. In addition to these results, we used the values that allow estimating the effect of the CR program on the physical and mental components of the participants up to 6 months after a coronary cardiac event. The values of Standard Error and Difference in Means (MD) (95% confidence intervals) with a statistical significance of *p* < 0.05 were then used for this meta-analysis by applying the SF-12 in the different studies that evaluated the effectiveness of the CR program in the different domains. This generic PROM (SF-12) was used because it is the one that best applies to this study, in which the scoring yields two summary measures: the Physical Component Summary (PCS) and the Mental Component Summary (MCS).

The data obtained from the studies included in the meta-analysis were combined, and a statistical analysis was performed using a random effects model. This type of model allows for the effects of heterogeneity due to methodological differences in the studies included, the type of CR program, and a small number of participants in some studies, as well as robust statistical results [17]. The effect of heterogeneity can be seen with the relevant I squared (I^2^) and the statistically significant homogeneity test (*p* < 0.05).

The different variables of the included studies were compared at the time of assessment after the CR program. The effect size of this program on the HRQoL of participating patients was calculated for each of the studies according to the assessment at 6 months post-coronary event. A sensitivity analysis was performed to understand each study intervention’s effect size. Thus, it is possible to understand the significance of each study, where the MD of each study ranged from 1.60 to 6.70 in the physical domain and −7.81 to 8.83 in the mental domain. The publication bias of the studies that assessed the HRQoL at 6 months post-program was calculated by Egger’s test (using SPSS Version 28.0), and the respective bias was analyzed by the asymmetry in the funnel plot (statistical significance for *p* < 0.10) [18].

## 3. Results

**Figure 1:**
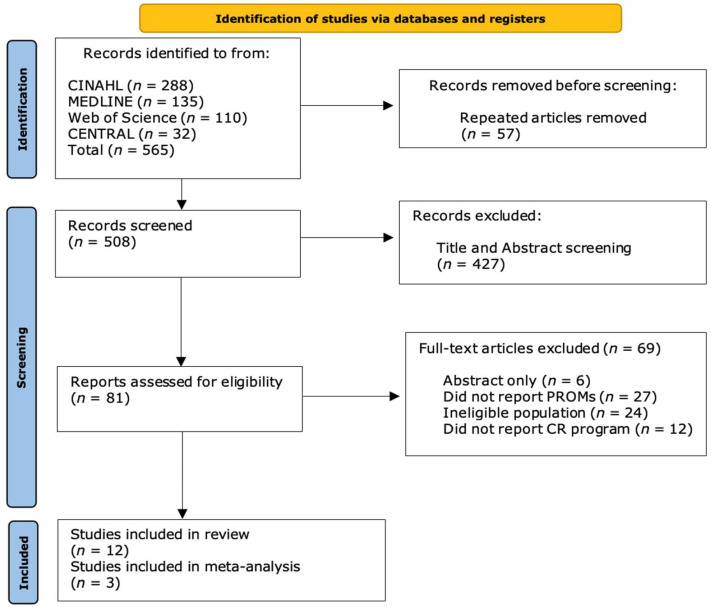
Flow diagram of the literature search of this meta-analysis (according to Preferred Reporting Items for Systematic Reviews and Meta-Analyses—PRISMA).

From the results obtained after the initial electronic search (*n* = 533), 476 articles were analyzed once duplicates were removed. From this extraction, after duplicate removal and evaluation of titles and abstracts, 80 articles were reviewed. A screening of articles based on full-text assessment according to the eligibility criteria was performed, resulting in 12 RCTs [19,20,21,22,23,24,25,26,27,28,29,30] that were included in the systematic review, and 3 of them [22,23,25] were considered suitable for meta-analysis to assess the physical and mental components of HRQoL (SF-12) at 6 months of follow-up, as shown in Figure 1.

### 3.1. Characteristics of Studies and Participants

The studies selected were conducted in China (*n* = 3) [21,23,26], Pakistan (*n* = 2) [25,29], Germany (*n* = 1) [30], and one study each in Italy [19], Egypt [20], Israel [22], the UAE [27], and the UK [24]. All studies had an RCT design comparing outcomes in two groups. One group participated in a structured CR program with intervention by a multidisciplinary team, which was compared with the other group that received usual care after coronary cardiac event treatment. In the different studies, the intervention lasted from 3 weeks to 12 months. According to the specifications of the intervention in the different studies, we can report the following: supervised individualized exercise training or group intervention, strength and resistance exercises (walking, treadmill, and stationary cycling), education intervention, and psychological support, as shown in Table 1.

Regarding the characteristics of the participants in this review, HRQoL was assessed in 2260 patients who participated in the CR programs after coronary cardiac event, with a mean age of 60.06 years. Of these participants, 69.29% were male, with one study reporting only men in the inclusion criteria.

### 3.2. Systematic Review

There is a variation in structure in some of the programs, which we note is typical of CR globally. However, in all studies selected for this meta-analysis, participants were supervised in exercise sessions throughout the intervention, and this was the most common type. Regarding follow-up, it took place in a mixed manner in most studies, i.e., at the health care facility and at home (telephonically). A CR program’s remaining session intervention (components) was delivered through health education sessions, medication management coaching, disease management, coaching to support self-efficacy through awareness of physiological and affective states, and social coaching.

Different PROMs were used throughout the follow-up of participants in the CR programs. The generic instruments used to assess HRQoL in the included studies were SF-12 (*n* = 5) [21,22,23,25,26], SF-36 (*n* = 1) [20], EQ-5D-3L (*n* = 1) [19], and EQ-5D-5L (*n* = 2) [24,30]. Those specific to cardiac conditions were the MacNew (*n* = 3) [24,25,29] and HeartQoL (*n* = 2) [27,28].

The results of our systematic review indicate a moderate overall quality of evidence, using the GRADE approach, when assessing HRQoL 6 months after intervention in a CR program (Supplementary Materials). There is a fair degree of confidence that clinical practice with multidisciplinary interventions in CR programs is associated with variations in HRQoL. There are some limitations to this review, including the heterogeneity of the study population, and different types of interventions: duration, frequency of physical training, education sessions, and social support. In addition, the very rigorous inclusion criteria in this analysis resulted in few studies on this topic, and therefore an improvement in other outcomes may not have been included.

### 3.3. Risk-of-Bias Assessment

The assessment of the risk of bias for the studies considered in our study was based on that recommended by Cochrane using the Rob2 tool [15]. After analysis of the studies, it was found that some did not detail methodological steps, and there were some considerations of possible bias (Supplementary Materials). Due to the clinical situation of each participant, it is sometimes necessary to adapt some aspects during the predefined program, with some risk of bias in terms of consistency in the implementation of the intervention planned in some studies [21,23,25,26,29]. The risk of reporting is low in all studies, as they presented the results of the PROMs used. The risk of bias in outcome selection is high in some studies [25,29], with some considerations in others [19,20,21,22,23,26,27,28]. This risk of bias is because the HRQoL assessment is through scales and questionnaires, and the authors did not indicate how this result was extracted. Overall, according to the risk of bias, more than 50% of the studies have low risk, and only 16.67% have high risk.

### 3.4. Meta-Analysis

**Figure 2:**
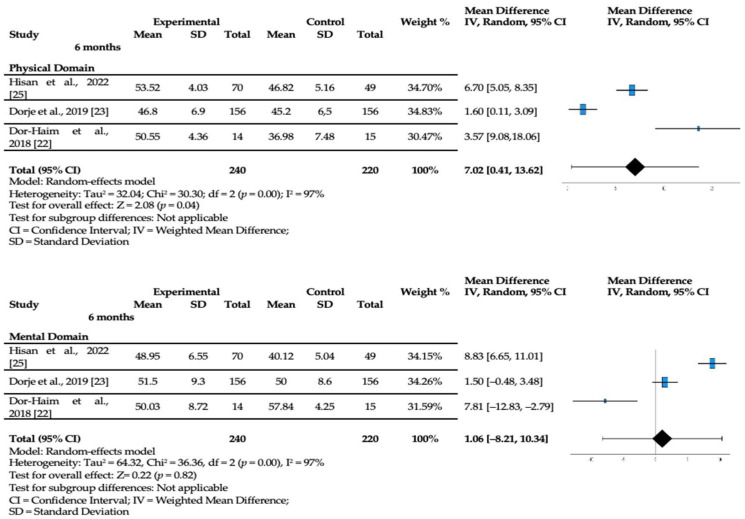
SF-12 domains at 6 months (Physical Component Summary and Mental Component Summary) [22,23,25].

The meta-analysis included studies that used PROMs to measure HRQoL. After the systematic review of the literature, the selected RCTs were used in the meta-analysis because they measured HRQoL over time after intervention in a CR program. Figure 2 presents the analysis of the RCTs, showing the quality of life of patients with coronary heart disease after intervention in a CR program.

In the three RCTs of the meta-analysis, there was a positive effect on quality of life at 6 months after starting the CR program. When assessed for up to 6 months of the program, there was statistically significant evidence (*p* = 0.04) showing an increase in the physical domain score (MD [7.02]; 95% CI [0.41, 13.62]) after the intervention. In the mental domain, although there were improvements in CR program participants, they were not statistically significant (MD [1.06]; 95% CI [−8.21, 10.34]). The asymmetry in the funnel plot can be verified by considering the studies included in the meta-analysis, showing a result with publication bias justified by the heterogeneity of the different studies (Supplementary Materials).

## 4. Discussion

The positive effect of CR programs on HRQoL, particularly the physical component at 6 months, highlights the benefits of these interventions. After applying the different PROMs to assess the HRQoL of participants with ischemic coronary disease in CR programs, and in this meta-analysis the SF-12, an improvement was shown in the physical dimension at 6 months after the start of the intervention. RCTs that assessed participants’ HRQoL after CR programs for coronary events using the same instrument were combined, thus measuring the effect of the intervention on the experimental group. Several generic and coronary-disease-specific PROMs were used to assess the HRQoL of participants in CR programs, highlighting the SF-12, SF-36, EQ-5D-5L, EQ-5D-3L, MacNew, and HeartQoL. There is a knowledge gap regarding the assessment of health status and quality of life reported by participants through PROMs in rehabilitation programs, and it is essential to demonstrate their importance [31,32,33,34,35,36,37]. Many of the RCTs on cardiovascular disease do not measure the components of HRQoL [38], knowing that it is critical for individualized care. It is known that only 23% of RCTs included HRQoL as a primary outcome and only 70% considered this outcome important, underutilizing PROMs in clinical decision making [38]. Recent reviews have concluded that it is challenging to compare HRQoL outcomes in participants in CR programs due to the interventions’ complexity and each patient’s clinical situation leading to the heterogeneity of the instruments [34,39]. Although several instruments were analyzed, we found that for HRQoL, the most used was the SF-12. This instrument is essential for assessing the HRQoL of patients with coronary heart disease who participate in CR programs, since it refers to aspects of well-being that are influenced by the physical and mental domains [40].

Regarding the timing of PROMs, most of the studies included in this review assessed HRQoL at baseline (at the beginning of the program) and over the following 6 months, even when the intervention period was conducted over a few weeks. A meta-analysis assessing HRQoL in patients with coronary syndrome showed significant improvements in physical performance and general health 6 months after applying different PROMs [13]. Monitoring the HRQoL of patients who participate in CR programs over time is important, since improvements are not only short term but also over a longer period due to the benefits of physical exercise. Physical exercise improves health and is one of the main components of CR programs [41]. Despite some heterogeneity, the studies included in this review included the dimension of physical exercise in the structure of the program carried out in a supervised manner at an early stage. Early-onset supervised exercise is positive because it improves physical function and exercise tolerance, allowing exercise to be continuously adapted to individual needs and abilities [42,43,44]. In this study, a positive effect on HRQoL was observed in the physical component 6 months after starting the program. In a review that studied the relationship of quality of life with exercise-based CR, the authors highlighted that the improvement in physical function translates into increased performance of activities of daily living, and patients with coronary disease reported higher scores in the assessment of their quality of life [13].

In the mental domain, the effect was also positive up to 6 months after starting the CR program compared to the control group, but without statistical significance. Anxiety and depression are common after acute myocardial infarction, may persist for a few months or even years, and may affect the dynamics and adherence to the CR program [44]. One of the studies conducted on quality of life in patients who suffered an acute myocardial infarction showed that these patients have a higher likelihood of negative psycho-emotional effects, leading to a deterioration in HRQoL [44]. Another study justifies this result, finding only a positive effect on the physical functioning of the participants [45].

In addition to the benefits for the mental and physical condition of patients who participate in CR programs, it is also important to mention in the discussion other benefits of implementing these programs. The health system also gains from the implementation of CR programs. Monitoring the outcomes associated with these programs in coronary pathology makes it possible to reduce the costs inherent in this syndrome and the associated burden and mortality [46]. The economic implications of these programs, compared to their benefits, are demonstrable and have a good cost-effectiveness ratio. A study of 601.099 patients ≥ 65 years hospitalized for coronary heart disease compared 5-year mortality in users and nonusers of CR, and it showed that CR improved mortality by 8.0% (*p* < 0.001) [47]. It also indicated that 1.344 QALYs (95% CI, 0.543–2.144) were added, showing improvements in quality of life and reducing the cost of treatment from USD 32,996 (95% CI, USD 21,942–66,494)/QALY to USD 30,188 (95% CI, USD 18,175–74,484)/QALY over a lifetime [47]. In fact, CR is a cost-effective treatment option for health services, and although it is underutilized, care providers should encourage patients to participate with the aim of improving quality of life and reducing health costs.

The main limitation of this meta-analysis is the limited number of studies, and some studies had small samples due to the complexity of the patient’s clinical condition and the known barriers to adherence to the programs. Another limitation is the inherent nature of cardiac rehabilitation interventions in the treatment group compared to the control group. Patients enrolled in the treatment arm may inadvertently receive multidisciplinary and more personalized care in some studies compared to their counterparts in the control group, potentially skewing outcomes.

## 5. Conclusions

Although the assessment of HRQoL in CR participants after coronary cardiac events is known, few studies include HRQoL as a primary outcome in the evaluation of the effect of CR. This study develops knowledge in CR by showing the different PROMs applied in the assessment of HRQoL and by demonstrating the importance of time in the assessment of the different components of HRQoL. This study shows the positive effect of CR on the physical and mental components of HRQoL after assessment by the same PROM, with the intervention of multidisciplinary teams. The importance of time in assessing HRQoL was demonstrated by the SF-12 during the follow-up period, with statistically significant results in the physical domain at 6 months.

Given the importance of these results, HRQoL improvement, regardless of the underlying disease or the CR program implemented, is important, and it is vital that health organizations implement PROMs by investing more time and resources in an implementation strategy.

## Figures and Tables

**Table 1:** Characteristics of included studies.

Author, Year	Country	Study Design (Sample Size)	Age (Years)	Type of Intervention	PROM(s): Measuring Quality of Life	Timing of PROM(s)
Campo et al., 2020 [19]	Italy	RCT (*n* = 235)	76 ^◊^	—IG ^6^ received 4 supervised sessions of exercise after discharge combined with home-based exercise. —CG ^1^ attended a health education intervention only.	EQ-5D-3L ^3^	Baseline, at month 6, 12
Casper et al., 2019 [20]	Egypt	RCT (*n* = 40)	52.3 ^◊^	—IG ^6^ received standard treatment, educational intervention 30–40 min/session every 2 weeks for 3 months, and regular telephone follow-up reminders every week for 3 months. —CG ^1^ attended counselling about diet and smoking cessation, adjusting cardiovascular medication when needed, and exercise intervention.	SF-36 ^8^	Baseline, at month 3
Deng et al., 2020 [21]	China	RCT (*n* = 70)	81.6 ^◊^	—IG ^6^ received a supervised session combined with home-based exercise; the duration was 12 weeks, 5 sessions/week with 40 min/session. —CG ^1^ received standard treatment including education, social support, and advice on diet and exercise.	SF-12 ^9^	Baseline, at month 3
Dor-Haim et al., 2018 [22]	Israel	RCT (*n* = 29)	47–69	—IG ^6^ received for 12 weeks a super-circuit training, performed moderate- to high-intensity exercise, alternating between resistance and aerobic training, at 75–85% of their heart reserve. —CG ^1^ participants performed exercise intervention at 60–70% of their cardiac reserve, where each session lasted 45 min.	SF-12 ^9^	Baseline, at month 3
Dorje et al., 2019 [23]	China	RCT (*n* = 312)	59.1 ^◊^	—IG ^6^ received a 2-month intensive program, followed by a 4-month step-down stage. During the intensive intervention phase, participants received 4 educational modules/week via chat. In the step-down phase, participants received only two cartoon pictures with key motivational messages per week. —CG ^1^ received standard care, as provided by their community doctors and cardiologists after hospital discharge.	SF-12 ^9^	Baseline, at month 2, 6, 12
Herring et al., 2021 [24]	UK	RCT (*n* = 291)	66.48 ^◊^	—IG ^6^ received one session with information leaflet and an education intervention that comprised 2 and 5 h sessions delivered 2 weeks apart by two trained facilitators. —CG ^1^ received one session with an information leaflet and returned to their general practitioner standard care.	EQ-5D-5L ^2^ MacNew ^7^	Baseline, at month 12
Hisam et al., 2022 [25]	Pakistan	RCT (*n* = 160)	53.6 ^◊^	—IG ^6^ received the intervention, supervised, in addition to standard post-ACS care. The first phase included individualized psychotherapy during the hospital stay and the second phase included diurnal mobile texting of standardized messages about healthy lifestyle changes through a specially developed app. —CG ^1^ received standard post-ACS care.	SF-12 ^9^ MacNew ^7^	Baseline, at month 3, 6
Ma et al., 2020 [26]	China	RCT (*n* = 300)	63.1 ^◊^	—IG ^6^ received the program, consisting of a four-part intervention: related health education, supervised exercise and surveillance, risk factor control, and psychological nursing. —CG ^1^ was given usual care.	SF-12 ^9^	Baseline, at month 3, 6, 12
Muthukrishnan et al., 2021 [27]	UAE	RCT (*n* = 24)	49 ^◊^	—IG ^6^ received an aerobic intervention with progressively intensive power walking based on a prescribed target heart rate on treadmill. —CG ^1^ received for 4 weeks a program,3 times a week, in 12 sessions under supervision.	HeartQoL ^5^	Baseline, at month 1
Pedersen et al., 2023 [28]	Denmark	RCT (*n* = 312)	49 ^◊^	—IG ^6^ received a multidisciplinary program and pedagogical strategy empowerment, motivation, and medical adherence. —CG ^1^ received a multidisciplinary program.	HeartQoL ^5^	Baseline, at month 6, 12
Ul-Haq et al., 2019 [29]	Pakistan	RCT (*n* = 206)	53 ^◊^	—IG ^6^ received a structured counselling intervention, counselling and health education, medicine prescription, and follow-up advice. —CG ^1^ obtained only the standard communication from the cardiologist and routine follow-up care.	GHQ ^4^ MacNew ^7^	Baseline, at month 8
Wienbergen et al., 2019 [30]	Germany	RCT (*n* = 281)	56.5 ^◊^	—IG ^6^ participants were educated and controlled to change their lifestyle in a way to reduce cardiovascular risk factors and maintain or optimize medical secondary prevention therapy (every 3 weeks the prevention assistant had personal telephone contact). —CG ^1^ received medical and interventional therapy following the current standard of care.	EQ-5D-5L ^2^	Baseline, at month 6, 12

^1^ CG—control group; ^2^ EQ-5D-5L—EuroQol5-dimension health outcome survey (5-level); ^3^ EQ-5D-3L—EuroQol5-dimension health outcome survey (3-level); ^4^ GHQ—General Health Questionnaire; ^5^ HeartQol—heart quality of life; ^6^ IG—intervention group; ^7^ MacNew—MacNew Heart Disease health-related quality of life questionnaire; ^8^ SF-36—Medical Outcomes Study 36-item Short-Form; ^9^ SF-12—12-item shortened version of SF-36; ^◊^ Indicates the mean age of the intervention group.

## Data Availability

The original contributions presented in the study are included in the article, further inquiries can be directed to the corresponding author.

## Supplementary Materials

The following supporting information can be downloaded at https://www.mdpi.com/article/10.3390/jcm13185576/s1.
