# Supplementary material for: Physical and Mental Components of Quality of Life after a Cardiac Rehabilitation Intervention: A Systematic Review and Meta-Analysis
Source: J Clin Med. 2024 Sep 20;13(18):5576. doi: 10.3390/jcm13185576 (PMC11433594; doi:10.3390/jcm13185576)
Supplement: Supplementary file 1 [file jcm-13-05576-s001.zip › jcm-3199646-supplementary.pdf]

Supplementary Figure (Funnel plot at 6 Months with 95% confidence intervals for change in HRQoL-physical domain)

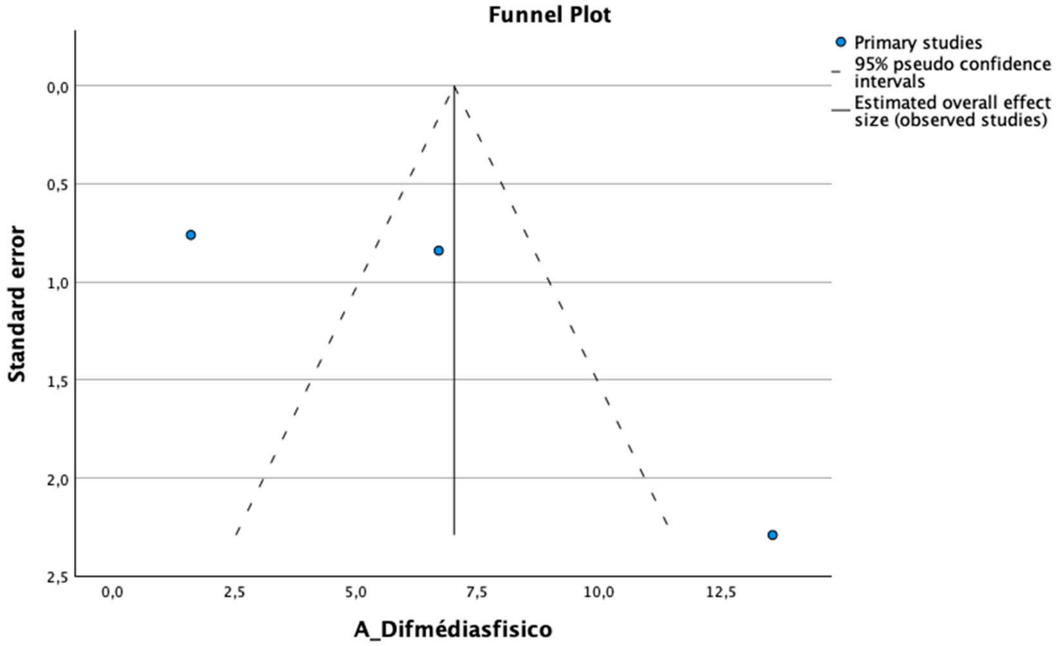

Supplementary Figure (Funnel plot at 6 Months with 95% confidence intervals for change in HRQoL-mental domain)

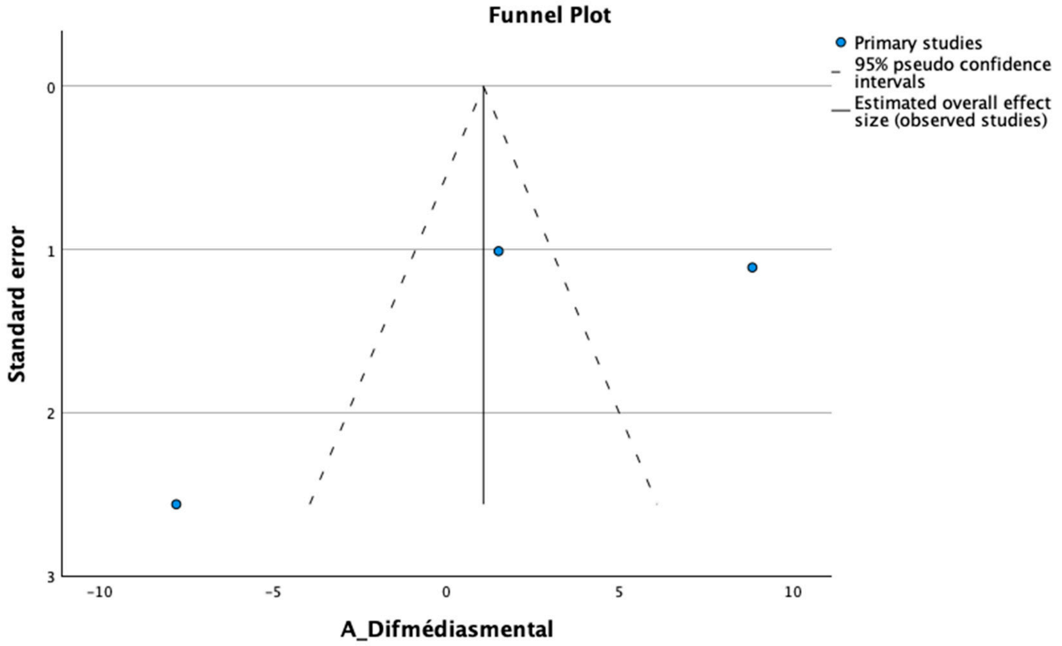

Supplementary Figure (GRADE assessment)

Unique ID	Experimental	Comparator	Outcome	Randomization process	Comment for randomization process	Deviations from intended interventions	Comment for deviations from intended	Missing outcome data	Comment for missing outcome data	Measurement of the outcome	Comment for measurement of the outcome	Selection of the reported result	Comment for selection of the reported result	Overall Bias
Dor-Haim	14	15	SF12	Low		Low		Low		Low		Some concerns		Some concerns
Dorje	156	156	SF12	Low		Low		Low		Low		High		Some concerns
Hisam	70	49	SF12	Some concerns		Some concerns		Low		Low		High		Some concerns
Ma	150	150	SF12	Low		Some concerns		Low		Low		High		Some concerns
Campo	118	117	EQ5D-3L	Low		Low		Low		Low		Some concerns		Some concerns
Casper	20	20	SF36	Low		Low		Low		Low		Some concerns		Low
Deng	35	35	SF12	Some concerns		Some concerns		Low		Low		High		High
Herring	145	146	EQ5D-5L; MacNewHRQoL	Low		Some concerns		Low		Low		Low		Low
Muthukrishnan	12	12	HeartQoL	Low		Low		Low		Low		Some concerns		Some concerns
Pedersen	156	156	MacNewHRQoL	Low		Low		Low		Low		Some concerns		Low
Ui-Haq	99	96	GHQ; MacNewHRQoL	Some concerns		Some concerns		Low		Low		High		High
Wienbergen	138	143	EQ5D-5L	Low		Some concerns		Low		Low		Low		Low

Unique ID	Study ID	Experimental	Comparator	Outcome	Weight	D1	D2	D3	D4	D5	Overall
Dor-Haim	NA	14	15	SF12	NA	+	+	+	+	!	!
Dorje	NA	156	156	SF12	NA	+	+	+	+	-	!
Hisam	NA	70	49	SF12	NA	!	!	+	+	-	-
Ma	NA	150	150	SF12	NA	+	!	+	+	-	!
Campo	NA	118	117	EQ5D-3L	NA	+	+	+	+	!	!
Casper	NA	20	20	SF36	NA	+	+	+	+	!	!
Deng	NA	35	35	SF12	NA	!	!	+	+	-	!
Herring	NA	145	146	EQ5D-5L; MacNewHRQo	NA	+	!	+	+	+	+
Muthukrishnan	NA	12	12	HeartQoL	NA	+	+	+	+	!	!
Pedersen	NA	156	156	MacNewHRQoL	NA	!	!	+	+	!	!
Ui-Haq	NA	99	96	GHQ; MacNewHRQoL	NA	!	!	+	+	-	-
Wienbergen	NA	138	143	EQ5D-5L	NA	+	!	+	+	+	+

\+ Low risk
! Some concerns
\- High risk

D1 Randomisation process
D2 Deviations from the intended interventions
D3 Missing outcome data
D4 Measurement of the outcome
D5 Selection of the reported result

Supplementary material (SF-12 questionnaire)

SF-12v2™ Health Survey

(SF-12 v2 Standard, US Version 2.0)

To be completed by the PATIENT

Identification Number

Event

Directions: *This survey asks for your views about your health. This information will help you keep track of how you feel and how well you are able to do your usual activities. If you need to change an answer, completely erase the incorrect mark and fill in the correct circle. If you are unsure about how to answer a question, please give the best answer you can.*

Today's Date (MM/DD/YY) __ __ / __ __ / __ __

Shade circles like this:
Not like this:

Mark only one answer for each question. Please do not mark outside the circles or make stray marks on the questionnaire.

	Excellent	Very Good	Good	Fair	Poor
**01. In general, would you say your health is:**	○	○	○	○	○

The following questions are about activities you might do during a typical day. Does your health now limit you in these activities? If so, how much?	Yes, limited a lot	Yes, limited a little	No, not limited at all
**02. Moderate activities, such as moving a table, pushing a vacuum cleaner, bowling, or playing golf**	○	○	○
**03. Climbing several flights of stairs**	○	○	○

During the past 4 weeks, how much of the time have you had any of the following problems with your work or other regular daily activities as a result of your physical health?	All of the time	Most of the time	Some of the time	A little of the time	None of the time
**04. Accomplished less than you would like**	○	○	○	○	○
**05. Were limited in the kind of work or other activities**	○	○	○	○	○

During the past 4 weeks, how much of the time have you had any of the following problems with your work or other regular daily activities as a result of any emotional problems (such as feeling depressed or anxious)?	All of the time	Most of the time	Some of the time	A little of the time	None of the time
**06. Accomplished less than you would like**	○	○	○	○	○
**07. Did work or activities less carefully than usual**	○	○	○	○	○

	Not at all	A little bit	Moderately	Quite a bit	Extremely
**08. During the past 4 weeks, how much did pain interfere with your normal work (including both work outside the home and housework)?**	○	○	○	○	○

These questions are about how you feel and how things have been with you during the past 4 weeks. For each question, please give the one answer that comes closest to the way you have been feeling. How much of the time during the past 4 weeks...	All of the time	Most of the time	Some of the time	A little of the time	None of the time
**09. Have you felt calm and peaceful**	○	○	○	○	○
**10. Did you have a lot of energy**	○	○	○	○	○
**11. Have you felt downhearted and depressed**	○	○	○	○	○
**12. During the past 4 weeks, how much of the time has your physical health or emotional problems interfered with your social activities (like visiting friends, relatives, etc.)?**	○	○	○	○	○
